# Development of recurrent facial palsy during plasmapheresis in Guillain-Barré syndrome: a case report

**DOI:** 10.1186/1752-1947-4-253

**Published:** 2010-08-06

**Authors:** Mary L Stevenson, Louis H Weimer, Ilya V Bogorad

**Affiliations:** 1 P & S Box 485, 630 West 168th Street, New York, NY 10032, US; 2 Neurological Institute of New York, Columbia University Medical Center, 710 W. 168th Street, Unit 55, New York, NY 10032, US; 3 91 Highgate Street, Needham, MA 02492, US

## Abstract

**Introduction:**

Guillain-Barré syndrome is an immune-mediated polyneuropathy that is routinely initially treated with either intravenous immunoglobulin or plasmapheresis. To the best of our knowledge, no association between plasmapheresis treatment and acute onset of facial neuropathy has been reported.

**Case presentation:**

A 35-year-old Caucasian man with no significant prior medical history developed ascending motor weakness and laboratory findings consistent with a diagnosis of Guillain-Barré syndrome. Plasmapheresis was initiated. Acute facial palsy developed during the plasma exchange that subsequently resolved and then acutely recurred during the subsequent plasma exchange.

**Conclusion:**

To the best of our knowledge, no prior cases of acute facial palsy developing during plasmapheresis treatment are known. Although facial nerve involvement is common in typical Guillain-Barré syndrome, the temporal association with treatment, near-complete resolution and later recurrence support the association. The possible mechanism of plasmapheresis-induced worsening of peripheral nerve function in Guillain-Barré syndrome is unknown.

## Introduction

Guillain-Barré syndrome (GBS) is an immune-mediated acute polyneuropathy typically characterized by ascending weakness and areflexia. An association with *Campylobacter jejuni *infection is most common; however, numerous associations are known [1]. Now recognized as a heterogeneous syndrome, different variants exist including demyelinating and axonal forms; the demyelinating variant is most common in the USA. Based on considerable clinical trial evidence, the American Academy of Neurology currently recommends treatment with either intravenous immunoglobulin (IVIG) or plasmapheresis within two to four weeks [2]. Although the disease may continue to advance during treatment, acute focal worsening is not a recognized treatment complication. We report a case of acute facial palsy that developed during plasma exchange, subsequently resolved, and then acutely recurred during the subsequent plasma exchange.

## Case presentation

A 35-year-old Caucasian man, with no significant prior medical history, developed symmetric ascending weakness and paresthesia. Six days prior to admission he noted bilateral foot and then posterior leg numbness. Chiropractic manipulation provided no relief. Two days later, he developed progressively ascending lower extremity weakness and increasing leg and foot tingling and numbness. Hand weakness and paresthesia began two days prior to admission and this spread to involve his shoulder girdle. His gait became unsteady, prompting him to come to our emergency department. Prior to admission, he also noted shortness of breath with exertion but not while at rest, feeling as though his heart was racing during exertion, and night sweats, all of which were unusual for him. He was an avid runner prior to the onset of his symptoms. His wife is a nurse practitioner and her home physical examination was described to show areflexia. He had no notable respiratory or gastrointestinal viral prodrome prior to the onset of his neurological symptoms. However, he described a mild, transient occipital, throbbing headache one morning at the start of his symptoms that resolved within two hours of onset. One day prior to admission, he noted mid-tongue numbness. Sensation in his extremities seemed altered and he had difficulty distinguishing hot from cold objects. His weakness and numbness were progressively worsening on the day of his admission. On the morning of admission he also noted one period of blurry vision, which spontaneously resolved within two hours. He denied nausea, diarrhea, dysuria, presyncope, vertigo, hearing loss, rash, diplopia, facial asymmetry, tremor, dysphagia, or dysarthria. He denied recent vaccinations.

On admission to our hospital, he had normal orientation and cognition. His extraocular movements were full without nystagmus. His visual fields were full; no papilledema was evident. His pupils reacted briskly without afferent defect. His facial strength and sensation was full and symmetric. Despite the subtle symptoms, his face was symmetric upon smiling, eyebrow wrinkling, lip pursing, and eye closure. Light touch and cold perception in his trigeminal nerve territories was normal. His hearing was intact. His palate elevated well, although he had an odd sensation in the back of his throat. His tongue was midline on protrusion. No dysarthria was noted. His limb strength demonstrated bilateral weakness ranging from Medical Research Council scale 4- to 4+/5 in his upper and lower extremities. Deep tendon reflexes were hypoactive in his arms and absent in both of his legs; he had decreased light touch, temperature, and pin-prick sensation bilaterally from his feet to his thighs, and in his hands ascending to his shoulders. No specific cerebellar abnormalities were evident. His gait was unsteady and wide-based and he displayed an inability to tandem walk.

Cerebral spinal fluid showed cytoalbuminologic dissociation with a protein of 51 mg/dL and two white blood cells per mm^3^. His serology was negative for IgG anti-GQ1b and anti-GM1 ganglioside and related antibodies. No human immunodeficiency virus antibodies were present. He had positive titers of cytomegalovirus IgG and IgM, and he had a borderline reactive cerebrospinal fluid Lyme antibody study though negative serum antibodies suggested a false positive result. Over the ensuing days, weakness continued to progress slowly in his arms and legs to a point at which he was no longer able to walk or raise his arms without difficulty. His face, however, remained uninvolved. No cranial sensory or motor deficits developed.

Plasmapheresis was initiated on day nine of his symptoms following insertion of a vascular catheter. Near the end of the first treatment, he developed severe right-sided facial weakness with dysgeusia, and an obvious facial droop appeared. The remainder of his neurological examination, including contralateral facial strength, remained unchanged. A brain magnetic resonance imaging (MRI) scan was performed two hours later and showed no restricted diffusion. This deficit completely resolved within thirty minutes and did not recur that day. Two days later, a second round of plasmapheresis was initiated. Calcium gluconate was given prior to the procedure because of mildly low-ionized calcium measures. Approximately half-way into the second treatment, his facial weakness reemerged, this time without resolution, and he developed a persistent right-sided facial droop, an asymmetric smile, and weak closure of the right eye. The plasma exchange was discontinued mid-treatment and he was closely observed. His forehead was asymmetric but notably less involved. His frontalis strength improved and was symmetric the day after this second round of plasmapheresis, though the remainder of his facial paralysis persisted.

Because of the association of recurrent acute facial weakness during plasmapheresis, the therapy was discontinued and a decision was made to substitute with a course of IVIG. He received a conventional dose of IVIG, which was a total dose of 2.0 mg/kg given as a 5-day treatment course (0.4 mg/kg per day of 6 percent IVIG). He tolerated the infusions without complication.

Despite treatment, the weakness in his extremities continued to slowly progress and he later developed left-sided facial weakness, first noted four days after his second plasmapheresis treatment. Additionally, on day 12 of his symptoms his difficulty chewing prompted a change to a soft mechanical diet; on day 14 of his course he failed a swallowing evaluation indicating probable pharyngeal weakness. His symptoms continued to progress and seemed to nadir by week five of his course.

Facial motor nerve conductions were performed on the day of the second plasmapheresis (day 11 of his symptoms). Normal distal latencies and normal and symmetric evoked amplitudes were found from common facial nerve stimulation and recording from his bilateral orbicularis oculi, nasalis, and orbicularis oris muscles. In all likelihood, insufficient time had elapsed for Wallerian degeneration to occur. Blink reflex studies demonstrated an increased R1 latency (16.1 ms) and absent ipsilateral and normal contralateral R2 responses following right-sided supraorbital stimulation. Left-sided stimulation demonstrated a mildly-increased R1 latency but normal ipsilateral and absent contralateral R2 responses.

Nerve conduction studies of his right median, ulnar, peroneal, and tibial nerves were performed on days nine, 18 and 26 of his course and showed a demyelinating pattern with axonal involvement that progressively worsened with each examination. Increased distal motor latencies, serially-reduced evoked motor amplitude, reduced sensory responses, and loss of F-waves ensued; conduction velocity remained relatively unaffected. Focal conduction block or significant temporal dispersion was not evident at any point. Abundant fibrillations were evident in multiple muscles in the studies performed on day 26.

His facial droop improved by the third week of his course, and continued to improve through week four. His face became symmetric. His clinical course was complicated by pneumonia, respiratory failure requiring intubation, and a tracheotomy. He was discharged on day 46 in a stable condition to an acute rehabilitation facility. At that point he had mild facial weakness, was able to symmetrically produce a small smile and could fully but not forcefully close his eyes.

One year later he has almost fully recovered, following extended rehabilitation and physical therapy, and he has returned to work. He continues to report numbness in his big toes, and partial numbness in his second and third toes bilaterally, with sporadic neuropathic pain occurring two to three times per week but not requiring the use of pain medications. His facial symptoms ultimately resolved.

## Conclusions

GBS typically produces relatively symmetric ascending weakness and depressed deep tendon reflexes or areflexia [3]. Plasmapheresis and IVIG are the mainstays of acute GBS treatment [2]. Conventional plasmapheresis is not recognized to induce acute worsening including facial neuropathy. Only one previous similar report, of two clinical cases, was identified. Chida *et al*. reported in 1998 two cases of bilateral facial palsy developing in Miller Fisher Syndrome, a GBS variant associated with GQ1b antibodies, which occurred in the setting of immunoadsorption plasmapheresis (IAP) therapy. In these cases, bilateral facial palsy developed after either three of three or three of five IAP treatments while other neurological deficits were improving [4]. IAP is a newer form of plasmapheresis that selectively removes IgG without removal of significant albumen and other blood components. It should be noted that the process does not remove notable amounts of IgM antibodies. This process has been shown to be efficacious in Miller Fisher Syndrome [5].

Our patient twice developed acute onset right-sided facial weakness during conventional plasmapheresis for GBS, with resolution of his symptoms in the first incidence and persistence of them in the latter. This close temporal association of his facial weakness onset is supportive of a direct relationship with the plasma exchange treatment. The remainder of his symptoms continued to gradually and slowly progress over days without acute changes. Plasmapheresis is proven to decrease the duration and severity of deficits [6]. It is believed that antecedent infection leads to the production of humoral and cellular immune effectors that cross-react with certain nerve or myelin epitopes [7]. More recent work involving immunoadsorption, which selectively removes specific antibodies, shows that IAP is also an efficacious treatment which removes specific anti-myelin antibodies associated with GBS [8,9]. Our case is highly suggestive of a direct relationship between plasma exchange and development of facial palsy. It is conceivable that protective antibodies were removed by this treatment leading to the acute facial neuropathy. Additionally, other unknown large molecular weight proteins serving to modulate the immune response may have been removed. The etiology of the peripheral nerve dysfunction is unknown at this stage. The mildly low-ionized calcium during the first exchange and calcium gluconate infusion during the second treatment is not likely a significant factor. Sudden improvement of neurological function is reported in some cases associated with plasma exchange; such improvement is thought to occur faster than that explicable by neuroregenerative processes such as remyelination. In these settings, antibody-mediated changes in ion channel function that restores neural transmission is proposed. Ultimately the affected side of the face had the same outcome as the later more conventionally-affected side. Plasmapheresis units should be watchful for acute changes in strength during exchange treatments that may be exacerbated by further treatment.

## Competing interests

The authors declare that they have no competing interests.

## Authors' contributions

MLS, IVB, and LHW reviewed the current literature and patient presentation to compile this case report. LHW analyzed the nerve conduction studies. All authors read and approved the final manuscript.

## Consent

Written informed consent was obtained from the patient for publication of this case report. A copy of the written consent is available for review by the Editor-in-Chief of this journal.
